# Intimate Partners’ Political Influence: Longitudinal Evidence for the Mutual Transmission of Party Support between Women and Men

**DOI:** 10.1177/01461672251360302

**Published:** 2025-08-26

**Authors:** Sam Fluit, Nickola C. Overall, Danny Osborne, Matthew D. Hammond, Chris G. Sibley

**Affiliations:** 1 School of Psychology, University of Auckland, New Zealand; 2 PROMENTA Research Center, Department of Psychology, University of Oslo, Norway; 3 School of Psychology, Victoria University of Wellington, New Zealand

**Keywords:** political socialization, political party support, political preferences transmission, romantic relationships, random intercepts cross-lagged panel model

## Abstract

Cross-sectional research demonstrates that intimate partners generally hold similar political preferences. It is unclear, however, whether intimate partners influence each other’s preferences over time. We address this oversight by investigating the transmission of political party support across 1,613 woman-man couples in long-term intimate relationships using 10 annual waves of longitudinal panel data. Actor-partner interdependence random intercepts cross-lagged panel models revealed that within-person increases in an intimate partner’s party support predicted within-person increases in the other couple member’s party support the following year. These partner effects emerged for six political parties in New Zealand and, with only one exception, did not differ in strength across women and men. Partner effects for self-rated political orientation were not significant. These results suggest that couples mutually reinforce each other’s political party support over time, advancing understanding of the interpersonal transmission of political preferences and identifying a dyadic process that may contribute to political polarization.

Increasing political polarization makes it important to understand the socialization of political preferences in adulthood (Hatemi & Ojeda, 2021; Sears & Brown, 2023), including how close others may influence political party support (Iyengar et al., 2018). Most prior work examining political socialization has focused on parent-child dyads, suggesting that children acquire political preferences—support for political parties and political attitudes—from their parents (see Jennings et al., 2009). Yet, intimate partners in couple relationships may be particularly influential in shaping political preferences in adulthood. A foundational longitudinal study spanning over 50 years by Alwin et al. (1991) highlights the power intimate partners may have to influence political preferences. Women from conservative families grew more politically liberal during their years at the liberal Bennington College and remained liberal decades later. Participants’ change toward liberalism was, however, mitigated—and sometimes reversed—for women who married conservative men.

Despite this seminal example, there is limited contemporary research examining the longitudinal transmission of political preferences in intimate relationships, including how the political preferences of intimate partners may influence both women’s and men’s preferences across time. In addition, prior work has primarily focused on the transmission of political preferences in two-party systems (Green et al., 2002; Hooghe & Boonen, 2015). The current study uses 10 annual waves of panel data to examine the transmission of political party support within 1,613 woman-man couples in New Zealand by testing whether within-person changes in an intimate partner’s party support are associated with within-person changes in the other couple member’s party support the following year. Examining intimate partner transmission within a multi-party and coalitional government system (the most prevalent form of democratic governing globally) allowed us to test whether intimate partners have a longitudinal influence on each other’s political party support across six distinct parties.

## Transmission of Political Preferences: Existing Theoretical and Empirical Work

The political socialization literature has traditionally focused on parents’ influence on children’s political preferences. Drawing upon the principles of observational learning (Bandura, 1986), political preferences and related behaviors are theorized to develop via the modeling of socialization agents, such as children observing their parents’ political behavior and discussions (Durmuşoğlu et al., 2023; Plutzer, 2002; Sears & Valentino, 1997). Political socialization in pre-adulthood occurs in various social environments, including the family (Van Ditmars, 2023), schools, and friendship networks (Settle et al., 2011). However, most political socialization work has examined whether children adopt their parents’ political preferences by assessing similarities across parents’ and children’s political preferences (see Sears & Brown, 2023, for a review).

The seminal Youth-Parent Socialization Panel in the United States illustrates the standard approach to studying political socialization. Jennings and Niemi (1968) and Jennings et al. (2009) surveyed parents (Generation 1), their children (Generation 2), and the next generation of children (Generation 3) on various political outcomes (e.g., vote choice, party identification, political trust). Parents and their children demonstrated considerable similarity in political preferences: Political preferences were moderately correlated between Generations 1 and 2 parent-child dyads in the 1960s, as well as between Generations 2 and 3 parent-child dyads in the 1990s. Many other studies, typically conducted in the United States or United Kingdom, identify similar cross-sectional associations across parents’ and children’s political preferences (see Durmuşoğlu et al., 2023; Hatemi & Ojeda, 2021).

Although political preferences were initially thought to be acquired in childhood and remain stable throughout adulthood (Franklin et al., 2004), political preferences change throughout the life course (Sears & Brown, 2023). Accordingly, political socialization processes should also involve adult-to-adult transmission in close relationships. Specifically, interactions with close others who support established beliefs should reinforce adults’ political preferences, whereas repeated interactions with close others who hold contrary beliefs should weaken established beliefs (Lazer et al., 2010). People are motivated to hold beliefs similar to those of close others because a “shared reality” fosters a sense of belonging and security (Hardin & Conley, 2001; Hardin & Higgins, 1996). Indeed, Jost et al. (2008) propose that people create and protect shared realities with others by adopting political ideologies and attitudes that foster mutual understanding, and by deemphasizing dissimilar attitudes that threaten desired relationships. In addition, political disagreements can create psychological stress (Huckfeldt et al., 2004), particularly when occurring within close relationships that people want to maintain. Thus, people may be motivated to change their political preferences to avoid or resolve conflicts.

Intimate relationships involve both frequent interactions and relationship maintenance motivations, making them a critical context for the transmission of political preferences. These relationships also feature repeated face-to-face communication with highly valued conversation partners, amplifying the exposure to, and impact of, others’ political preferences (Alwin et al., 1991; Beck, 1991; Coffé & Need, 2010). Indeed, adults report intimate partners as their most important discussants for political content, surpassing other valued relationships including those with parents, children, friends, co-workers, and acquaintances (Carlson et al., 2020). Moreover, the high levels of closeness inherent in intimate relationships should magnify the need to generate and maintain a shared reality with intimate partners, as well as motivate intimate partners to reduce disagreements and tensions caused by diverging political preferences. Accordingly, several studies, predominantly in the United States and United Kingdom, identify moderately sized positive cross-sectional associations among intimate partners’ political party identification and political orientation (Coffé & Need, 2010; Gordon et al., 2024; Kan & Heath, 2006; Lampard, 1997; Leikas et al., 2018; Mancosu & Vezzoni, 2018). Associations between partners’ political preferences appear somewhat stronger in intimate relationships of longer duration (Alford et al., 2011; Jennings et al., 2009; Zuckerman et al., 2005), suggesting these associations might reflect transmission across intimate partners that accumulates over time.

## Advancing Understanding of Transmission of Political Preferences in Intimate Relationships

Despite growing theoretical and empirical work suggesting that political preferences may be transmitted within intimate relationships, the existing studies primarily involve cross-sectional associations that do not provide evidence of whether intimate partners’ influence each other’s political preferences across time. Moreover, although a few studies have adopted a longitudinal approach (e.g., Alwin et al., 1991; Iyengar et al., 2018; Kuhn, 2009; Stoker & Jennings, 2005; Zuckerman et al., 2005), these studies have concentrated on within-wave similarities rather than changes in political preferences across time (Alwin et al., 1991; Stoker & Jennings, 2005). One notable study provided stronger longitudinal evidence. In a U.K. sample of 1,428 woman-man couples, Zuckerman et al. (2005) found that intimate partners’ support for the Conservative or Labour Party at one time point predicted the other couple members’ support for the Conservative or Labour Party at the next time point. In the same study with a German sample of 1,033 woman-man couples, these partner effects replicated: Partners’ support for the Christian Democrats or Social Democrats at one time point predicted the other couple members’ support for that party at the next time point. Zuckerman et al. thus concluded that “the story is simple: people respond directly to the partisanship of their spouses and partners” (p. 91).

Though informative, the existing longitudinal studies provide relatively weak evidence that partners influence each other’s political preferences because they use methods that confound between-person processes with within-person change. For example, both an individual (i.e., actor) and their intimate partner (i.e., partner) may strongly support a given political party across assessments. However, these trait-like, between-person effects do not tell us whether within-person changes in a partner’s party support at one assessment predict similar within-person changes in an actor’s party support at a subsequent assessment. Such questions require the separation of between-person differences (i.e., trait-like, stable differences in party support) from within-person changes (i.e., deviations from a person’s trait-level party support at a given assessment occasion). The random intercepts cross-lagged panel model (RI-CLPM; Hamaker et al., 2015) provides these insights and offers an elegant approach to examine within-person change (Osborne & Little, 2024). Of particular relevance to the question of whether political preferences are transmitted across intimate partners, RI-CLPMs test whether within-person changes in a partner’s variable (e.g., partner’s political preferences) at a given timepoint are associated with within-person changes in the actor’s variable (e.g., actor’s political preferences) at a subsequent timepoint.

Assessing longitudinal within-person change in political preferences across adult intimate partners also uniquely enables the study of bidirectional effects (Mancosu & Vezzoni, 2018). Most political socialization research has assessed unidirectional political transmission from parent to child (see Hatemi & Ojeda, 2021), but political socialization should be a mutual, two-way process. Given prior assumptions of differences between women and men (e.g., see Kan & Heath, 2006), we distinguish between woman and man dyad members to test for bidirectional influences—a research question that has rarely been examined. Stoker and Jennings (2005) examined whether husbands influence their wives’ political preferences more strongly than wives influence husbands’ preferences. Husbands had significantly stronger effects on wives’ preferences in only 5 out of 21 tests. Although wives did not have a stronger effect on husbands’ preferences in any tests, it is notable that most tests revealed no gender differences. Kan and Heath (2006) also found cross-sectional evidence among 1,423 U.K. couples that the effect of intimate partners’ support for the Conservative Party and Liberal Democrats was equal across women and men. In the current study, we use a large sample and separate between-person differences and within-person changes to more robustly examine whether women and men in intimate relationships influence each other’s political preferences over time.

## Current Study

The current study provides three important advancements to the political socialization literature. First, we expand the nearly exclusive focus on the unidirectional transmission of political preferences in parent-child dyads to examine the bidirectional transmission of political preferences in a crucial context for political socialization in adulthood: intimate partner relationships. Second, we uniquely employ statistical methods and many waves of longitudinal dyadic data to assess whether within-person changes in intimate partners’ political preferences predict within-person changes in actors’ political preferences across time. Third, we test potential gender differences in the longitudinal transmission of political preferences across intimate partners.

To make these advances, we utilized 10 annual waves of data from the New Zealand Attitudes and Values Study (NZAVS), a longitudinal national probability panel study. For the current study, we identified and analyzed data from 1,613 unique woman-man couples who completed the assessments of interest (number of couples varied per wave, see Table 1). We separated between-person differences and within-person changes among couples by combining techniques from actor-partner interdependence models (APIM) and RI-CLPMs, thus estimating RI-CLPMs that integrate two people (Hamaker et al., 2015; Kenny et al., 2020). This approach allowed us to test whether within-person increases (or decreases) in a *partner’s* party support predicted subsequent within-person increases (or decreases) in an *actor’s* party support. We also tested whether these partner effects significantly differed across women and men.

**Table 1.** Number of Woman-Man Couples per Wave and Demographic Characteristics of Women and Men Across 10 Consecutive Years of Data Collection.

		Mean (*SD*)				(%)			Ethnicity (%)			
Time	*N*	Age	Education	SEI	Rel. Length	Married	Parent	Religious	European	Māori	Pacific	Asian
*Women*												
Time 5	136	49.47 (12.89)	5.21 (2.82)	54.62 (15.73)	22.20 (13.32)	77.04	78.52	36.57	94.85	11.03	2.21	4.41
Time 6	112	52.08 (12.29)	5.47 (2.79)	55.48 (15.42)	25.36 (12.81)	82.14	81.25	35.71	94.64	12.50	2.68	4.46
Time 7	93	53.85 (12.81)	5.58 (2.73)	55.43 (14.52)	25.99 (13.59)	83.87	76.34	40.86	95.70	9.68	3.23	6.45
Time 8	199	51.69 (13.16)	5.67 (2.79)	56.48 (15.03)	24.82 (13.44)	80.30	77.78	42.64	95.46	7.58	4.04	5.05
Time 9	136	53.87 (11.87)	5.82 (2.78)	57.46 (13.32)	26.10 (12.79)	84.56	80.88	37.50	94.85	11.03	4.41	5.15
Time 10	874	50.17 (11.58)	5.80 (2.71)	56.23 (15.40)	22.13 (12.58)	80.14	80.09	41.70	93.71	8.24	2.52	5.26
Time 11	813	53.96 (11.80)	6.22 (2.65)	57.92 (14.50)	24.12 (13.24)	80.52	80.17	35.51	96.18	6.65	2.83	3.45
Time 12	950	54.22 (12.25)	6.35 (2.53)	58.04 (14.88)	25.17 (13.44)	80.42	81.56	35.20	95.36	7.38	3.16	5.06
Time 13	721	56.67 (12.24)	6.41 (2.55)	58.38 (14.26)	27.24 (14.01)	81.93	81.06	34.82	96.53	5.83	2.50	4.03
Time 14	677	57.71 (12.41)	6.36 (2.61)	58.26 (13.12)	28.92 (14.07)	82.33	83.14	36.13	96.75	6.80	1.92	3.84
*Men*												
Time 5	136	52.17 (14.06)	5.44 (2.76)	54.62 (15.73)	22.16 (13.16)	77.04	84.45	37.04	96.32	7.35	2.21	2.94
Time 6	112	54.72 (13.55)	5.79 (2.72)	55.48 (15.42)	25.36 (12.78)	82.14	85.72	35.71	96.43	10.71	0.89	3.57
Time 7	93	56.54 (14.58)	5.73 (2.64)	55.43 (14.52)	25.96 (13.25)	83.87	83.87	36.56	97.85	8.60	2.15	3.23
Time 8	199	53.42 (14.20)	5.69 (2.57)	56.48 (15.03)	24.30 (13.39)	80.20	80.81	36.04	96.47	10.10	3.03	3.54
Time 9	136	55.90 (12.94)	5.87 (2.50)	57.46 (13.32)	26.11 (12.77)	84.56	86.03	36.03	96.32	11.76	2.94	4.41
Time 10	874	51.98 (12.13)	5.46 (2.68)	56.23 (15.40)	22.16 (12.80)	80.30	81.2	33.64	94.17	10.07	2.29	4.23
Time 11	813	55.91(12.25)	5.91(2.64)	57.92(14.50)	24.12(13.24)	80.42	82.25	29.75	97.17	7.14	1.85	2.59
Time 12	950	56.13 (12.73)	6.10 (2.56)	58.04 (14.88)	25.10 (13.59)	80.59	83.35	27.71	96.31	6.43	1.79	2.85
Time 13	721	58.66 (12.75)	6.26 (2.43)	58.38 (14.26)	27.20 (14.05)	82.70	83.54	26.98	97.92	6.11	1.39	2.08
Time 14	677	59.75 (13.07)	6.25 (2.50)	58.26 (13.12)	28.63 (14.10)	82.71	85.38	30.45	97.49	4.87	1.48	1.77

*Note.* Education was measured by highest level of qualification, ranging from 0 (*no qualification*) to 10 (*doctorate*; for reference 5 = *certificate/diploma*; 6 = *bachelor’s degree*). Participants could select multiple ethnicities. *SD* *=* standard deviation; SEI = socio-economic index, which is an occupation-based measure of socioeconomic position ranging from 10 to 90; Rel. Length = relationship length in years.

We examined whether the longitudinal associations assessing intimate partners’ influence on political party support were evident across six distinct parties. New Zealand has a multi-party democratic system, where governments are often formed between coalitions of multiple parties. Accordingly, the NZAVS assesses support for six political parties at each wave, including the two largest parties—the center-right National Party and the center-left Labour Party—and four smaller parties focusing on environmental issues (the Green Party), nationalism/populism (the New Zealand First [NZ First] Party), Indigenous rights (Te Pāti Māori), and libertarianism (the Association of Consumers and Taxpayers [ACT] Party; Lehmann et al., 2023). Unlike most prior studies, which have assessed political preferences in the United States’ two-party system or support for two dominant parties in the United Kingdom, measuring support for multiple political parties allowed us to test whether the effect of partners on within-person changes in actors’ political party support was evident across six distinct parties.

Finally, although our primary focus was on the multiple measures of party support, the NZAVS also includes a single self-rated measure of political orientation (liberal to conservative; Jost, 2006). Thus, we ran additional analyses to assess whether within-person changes in partners’ political orientation predicted subsequent within-person changes in actors’ political orientation.

## Methods

### Procedure and Participants

The NZAVS is an ongoing nationwide panel study that began in 2009 based on a random sample of the electoral roll and expanded in subsequent years via booster sampling. Participants are randomly sampled and invited to complete an annual postal or web-based questionnaire. Prior to Time 10, some participants’ partners asked whether they could also join the study. The couple sample thus began via self-selection. From Time 10 onwards, the questionnaire included an advertisement encouraging participants who were in a relationship to invite their partner to join. The NZAVS uses a strict method to identify couples (also see Lee et al., 2020; Waddell et al., 2025). First, we identified participants who shared a postal address (or a landline or email address if one person provided a residential address and the other provided a postal box in the same region). For cohabiting pairs to be identified as a couple, both participants needed to report at least one of the following characteristics: (a) same relationship type and similar relationship duration, (b) if married, same date of marriage, and (c) the gender of both participants consistent with their reported sexual orientation. If there were more than 12 years difference in age (increasing the possibility of being a parent-child dyad), then participants had to have the (a) same date of marriage or (b) same birthdate of one or more children.

Given that few couples were identified in the earlier smaller waves of the NZAVS (for an overview of the number of couples per wave, see Sibley, 2025), data from Time 5 (2013) to the most recent Time 14 (2022) were included to ensure that there was a sufficient number of couples for model estimation. These inclusion criteria resulted in a sample of 1,613 woman-man couples. Given that the sample size was determined by the number of couples who participated in the NZAVS, a priori power analyses were not conducted. In addition, no prior studies matched our design close enough to provide reasonable estimates for power analyses. Thus, we conducted Monte Carlo simulations (with 5,000 repetitions) to assess power (Muthén & Muthén, 2002). We used the parameters for the model assessing support for the Labour Party, which contained the smallest cross-lagged partner effect for women (i.e., *b* = 0.065). These simulations revealed that the study would reproduce a partner effect of *b* = 0.065 100% of the time.

Table 1 summarizes the number of couples at each wave, which ranged between 93 and 950 couples per wave (*M* = 471 couples; *M*_completion_ = 2.86 waves for men and 2.81 waves for women). Full Information Maximum Likelihood allows for missing data and estimates each aspect of the model using all available data (Enders & Bandalos, 2001). All cross-lagged parameters were thus estimated based on all available data from couples who had completed the given wave, with couples who completed more waves contributing more to the parameter estimates.

Table 1 also provides demographic data at each wave. On average, participants were in their 50s and involved in long-term relationships. Most couples were married (*M* = 81.12%). Relationship length at each wave is reported in Table 1. The mean relationship length across 10 waves of data collection, weighted on the number of couples at each wave, was *M* = 25.19 years (standard deviation [*SD*] = 13.42 years). Additional analyses controlling for relationship length did not alter the results (see Table S1 in the Online Supplemental Materials [OSMs]). Average education levels across waves (5.21–6.41) suggest that, on average, women and men had some form of high school certificate/diploma or bachelor’s degree (6 = *bachelor’s degree*). Most participants were parents, identified as NZ European, and around a third were religious.

### Measures

#### Political Party Support

At all timepoints, participants were instructed to “Please rate how strongly you oppose or support each of the following political parties”: the National Party, the Labour Party, the Green Party, Te Pāti Māori, NZ First, and the ACT Party (Sibley & Wilson, 2007). Response options ranged from 1 (*strongly oppose*) to 7 (*strongly support*). A higher score reflected greater support for the given political party.

#### Political Orientation

Participants were also asked to “Please rate how politically liberal versus conservative you see yourself as being” (Jost, 2006) on a 1 (*extremely liberal*) to 7 (*extremely conservative*) scale. Higher scores reflect a more conservative orientation.

#### Demographic Variables

Gender was coded from an open-ended question into three categories: woman = 1, gender diverse = 0.5, man = 0. Given our aim to test intimate partner transmission among woman-man couples, our analyses focused on participants who identified as either woman or man. Participants identified as NZ European, Māori, Pacific, and Asian (participants could choose multiple ethnicities). Socio-economic index was used as an occupation-based measure of socioeconomic position (Boven et al., 2021), which ranges from 10 to 90; higher scores indicate a higher socioeconomic position. Education was measured by asking participants to report their highest level of qualification. Responses ranged from 0 (*no qualification*) to 10 (*doctorate*) and were coded consistent with New Zealand Qualifications Authority (2012). Parenthood status was coded from participants’ reports of how many children they had parented. Finally, religiosity was assessed by asking whether participants identified “with a religion and/or spiritual group” (0 = *no*, 1 = *yes*).

### Analysis

We estimated six APIMs within the RI-CLPM framework to model within-person changes in political party support within and across women and men in intimate relationships while accounting for stable between-person differences in political party support across time. These APIMs represent analyses of distinguishable dyads in which a key theoretically relevant factor—gender—distinguishes across members in woman-man couples (Kenny et al., 2020). Distinguishable APIMs specify an actor and partner effect for women and an actor and partner effect for men, with the resulting estimates representing average effects across couples in the sample. The models also allow tests of whether these average actor and partner effects are of similar strength or significantly differ across gender. APIMs within the RI-CLPM framework further assess within-person actor and partner effects after accounting for similarities in stable between-person differences across women and men couple members.

**Figure 1.**
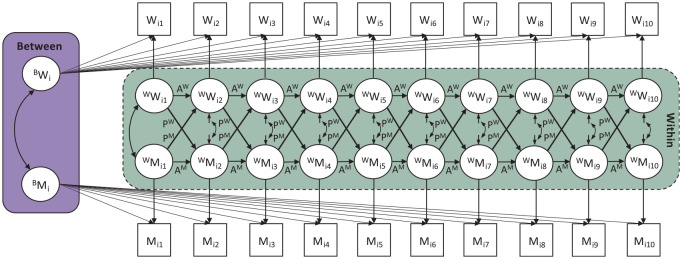
Actor-partner interdependence RI-CLPM assessing the transmission of political preferences between women and men in intimate relationships. The repeated assessments of political party support across 10 annual waves of the NZAVS for women (W_i1–10_) and men (M_i1–10_) were used to specify stable between-person levels of political party support for women (^B^W_i_) and men (^B^M_i_), as well as within-person deviations from between-person averages at each time point for women (^W^W_i1-i10_) and men (^W^M_i1-i10_). The cross-lagged paths (marked P^W^; effect of women partners on men actors, and P^M^; effect of men partners on women actors) estimate the extent to which within-person deviations in intimate partners’ political party support predicted within-person deviations in actors’ political party support the following year after adjusting for the similarity across partners within the same dyad (*i*) in average levels and within-person deviations at each time point (curved arrows), and the carry-over of within-person deviations across time within each person (paths marked A). RI-CLPM = random intercepts cross-lagged panel model; NZAVS = New Zealand Attitudes and Values Study.

The conceptual model is displayed in Figure 1. First, assessments of political party support across the 10 waves were modeled as equal estimates of a random intercept specifying mean levels of party support across time for women (^B^W_I_) and men (^B^M_I_) couple members. As shown in the “Between” section of Figure 1, the covariance between random intercepts of women (^B^W_I_) and men (^B^M_I_) was modeled to account for similarity in between-person levels of political party support across women and men couple members.

Second, each assessment of political party support across the 10 waves was also used to specify within-person deviations from each woman’s (^W^W_i1–10_) or man’s (^W^M_i1–10_) random intercept, which reflect the difference between the observed assessment at the given time point and the expected score based on the person’s between-person mean. As shown in the “Within” section of Figure 1, the autoregressive paths for women (A^W^) and men (A^M^) represent actor effects—the extent to which within-person deviations in women’s or men’s levels of political party support in one year are associated with similar within-person deviations in their political party support in the subsequent year. Positive autoregressive effects would indicate that, on average, within-person increases (or decreases) from people’s mean political party support at a given timepoint are associated with within-person increases (or decreases) at the next time point. Negative autoregressive effects would signify that, on average, within-person increases (or decreases) are associated with within-person decreases (or increases) at the next time point.

The cross-lagged effects across women’s and men’s within-person components (P^W^ and P^M^) represent partner effects and test our main research question. P^W^ tests the effect of women partners’ party support on within-person changes in men actors’ party support by estimating the degree to which within-person deviations in women partners’ political party support predict within-person deviations in men actors’ party support the following year. P^M^ estimates the degree to which within-person deviations in men partners’ political party support predict within-person deviations in women actors’ political party support the following year.

Positive cross-lagged effects would indicate that, on average, the more women (or men) partners exhibited a within-person increase (or decrease) from their average political party support at one time point, the more men (or women) actors exhibited a within-person increase (or decrease) at the next time point. Negative cross-lagged effects would indicate that the more women (or men) partners exhibited a within-person increase (or decrease) from their average political party support at one time point, the more men (or women) actors exhibited a within-person decrease (or increase) at the next time point.

We did not expect the autoregressive (actor) and cross-lagged (partner) paths to vary across assessments (see Osborne & Little, 2024). To test this assumption, we conducted a series of nested models: (a) fully unconstrained nonstationary models that allowed both autoregressive (actor) and cross-lagged (partner) paths to vary across waves, (b) partially stationary models that constrained autoregressive (actor) paths to be equal across waves but allowed the cross-lagged (partner) paths to vary, and (c) fully stationary models that constrained both autoregressive (actor) and cross-lagged (partner) paths to be equal across waves. Results from all models are reported in the OSM, Table S2. The stationary models provided the best model fit and represent the most parsimonious solution for all outcomes tested. Thus, in the primary analyses, we constrained the autoregressive (actor) and cross-lagged (partner) paths to be equal across waves. M*plus* syntax is available at https://osf.io/75snb/wiki/home/. This study and these analyses were not preregistered. We report all measures used, and all analyses conducted, for this study in the paper.

## Results

Table 2 reports the descriptive statistics of political party support for each wave. Across waves, average support for the two major parties—the center-right National Party and the center-left Labour Party—was mostly above the midpoint of the scale. The minor party that focused on environmental issues (i.e., the Green Party) received support comparable to the two major parties. Participants were generally less supportive of the smaller parties focusing on nationalism/populism (NZ First) and libertarianism (ACT Party), while the party focused on Indigenous rights (Te Pāti Māori) scored around the midpoint of the scale. Collectively, these data allowed us to examine political transmission across different parties, including change for more and less popular parties on average.

**Table 2.** Means and *SD*s (in Parentheses) of Political Party Support for Women and Men Over 10 Consecutive Years of Data Collection.

Time	*N*	National	Labour	Māori	ACT	Green	NZ First	Pol. Ori.
*Women*								
Time 5	136	4.23 (2.04)	3.92 (1.79)	3.12 (1.49)	2.57 (1.48)	4.30 (1.81)	2.63 (1.47)	3.55 (1.23)
Time 6	112	4.06 (2.15)	4.16 (1.78)	3.46 (1.51)	2.54 (1.48)	4.36 (1.89)	2.95 (1.61)	3.61 (1.37)
Time 7	93	3.93 (2.09)	4.00 (1.80)	3.42 (1.36)	2.56 (1.55)	4.43 (1.92)	2.84 (1.75)	3.74 (1.47)
Time 8	199	4.22 (2.00)	3.93 (1.63)	3.37 (1.51)	2.59 (1.41)	4.38 (1.84)	2.91 (1.65)	3.50 (1.39)
Time 9	136	4.15 (1.98)	4.55 (1.77)	3.57 (1.53)	2.57 (1.50)	4.48 (1.81)	2.84 (1.68)	3.45 (1.37)
Time 10	874	3.88 (1.93)	4.72 (1.72)	3.47 (1.57)	2.37 (1.47)	4.40 (1.78)	2.81 (1.51)	3.55 (1.39)
Time 11	813	3.47 (1.91)	4.86 (1.76)	3.62 (1.52)	2.56 (1.57)	4.55 (1.89)	2.83 (1.47)	3.39 (1.39)
Time 12	950	3.40 (1.81)	4.91 (1.79)	3.75 (1.58)	2.79 (1.75)	4.46 (1.90)	2.24 (1.37)	3.31 (1.39)
Time 13	721	3.45 (1.82)	4.41 (2.00)	3.54 (1.66)	2.93 (1.84)	4.16 (1.95)	2.28 (1.37)	3.39 (1.40)
Time 14	677	3.44 (1.92)	4.41 (1.98)	3.60 (1.74)	2.82 (1.91)	4.31 (2.00)	2.31 (1.44)	3.38 (1.43)
*Men*								
Time 5	136	4.19 (2.07)	3.67 (1.88)	2.84 (1.52)	2.37 (1.59)	3.49 (1.94)	2.54 (1.54)	3.66 (1.38)
Time 6	112	4.15 (2.06)	4.16 (1.64)	3.38 (1.39)	2.76 (1.62)	4.11 (1.92)	3.15 (1.61)	3.49 (1.53)
Time 7	93	4.07 (2.12)	4.00 (1.69)	3.17 (1.49)	2.53 (1.62)	3.86 (1.98)	2.94 (1.56)	3.42 (1.43)
Time 8	199	4.19 (1.95)	3.73 (1.66)	3.11 (1.50)	2.68 (1.65)	3.87 (1.99)	2.91 (1.74)	3.55 (1.49)
Time 9	136	3.88 (2.08)	4.26 (1.74)	3.23 (1.50)	2.53 (1.68)	3.93 (1.90)	2.99 (1.57)	3.51 (1.50)
Time 10	874	4.04 (1.92)	4.30 (1.70)	3.22 (1.58)	2.56 (1.60)	3.95 (1.88)	3.01 (1.55)	3.63 (1.41)
Time 11	813	3.71 (1.97)	4.45 (1.82)	3.34 (1.54)	2.80 (1.77)	4.06 (2.01)	2.84 (1.49)	3.42 (1.47)
Time 12	950	3.51 (1.85)	4.50 (1.81)	3.42 (1.66)	3.07 (1.95)	3.96 (2.08)	2.31 (1.37)	3.27 (1.43)
Time 13	721	3.74 (1.86)	4.02 (1.99)	3.18 (1.66)	3.14 (1.99)	3.76 (2.02)	2.38 (1.45)	3.49 (1.40)
Time 14	677	3.74 (1.87)	3.99 (2.03)	3.23 (1.74)	3.23 (2.09)	3.79 (2.07)	2.45 (1.47)	3.48 (1.49)

*Note. SD* *=* standard deviation; ACT = Association of Consumers and Taxpayers; NZ First = New Zealand First; Pol. Ori. = political orientation.

### Intimate Partner Transmission of Political Party Support

Our primary analyses assessed intimate partners’ influence on political party support by testing whether within-person changes in partners’ political party support predicted within-person changes in actors’ party support for both women and men. Separate models were estimated for each political party and provided distinct tests of whether the influence of intimate partners was evident across all six political parties. All models employed Bayesian estimation, and 95% credibility intervals (CIs) are reported. The point estimate represents the average effect across the sample, whereas the CI captures the probability that the actual population parameter lies within the given range of values. Significant results would thus suggest that, on average, changes in women and men partners’ political party support at one time point are associated with changes in actors’ political party support at the next assessment occasion.

Table 3 presents the correlations between women’s and men’s random intercepts for each political party. Significant correlations for all six parties (ranging from .506 to .745; *p*s < .001) revealed that women and men who expressed more support for a given political party on average were likely to have a partner who also expressed more support for that political party on average. Table 3 also presents the autoregressive or actor effects. The autoregressive paths were significant and positive for each political party, indicating that on average, within-person increases (or decreases) from women’s and men’s trait-level mean political party support at one time point were associated with within-person increases (or decreases) from their trait-level mean political party support the subsequent year.

**Table 3.** Bayesian Estimates of the Correlations Between Random Intercepts for Intimate Partners and the Unstandardized Autoregressive Associations for Political Party Support Across Time for Women and Men (*N*_couples_ = 1,613).

			A^W^		A^M^	
Outcome	*r*(^B^W_i_, ^B^M_i_)	95% CI	*b*	95% CI	*b*	95% CI
Labour	.725	[.689, .759]	0.348	[0.287, 0.409]	0.319	[0.254, 0.382]
National	.732	[.700, .762]	0.311	[0.244, 0.377]	0.223	[0.159, 0.294]
Māori	.563	[.502, .621]	0.208	[0.140, 0.278]	0.356	[0.297, 0.416]
ACT	.598	[.501, .674]	0.425	[0.369, 0.478]	0.561	[0.502, 0.618]
Green	.745	[.713, .774]	0.170	[0.107, 0.233]	0.291	[0.222, 0.360]
NZ First	.506	[.438, .570]	0.165	[0.104, 0.227]	0.256	[0.194, 0.316]
Pol. Ori.	.684	[.645, .720]	0.139	[0.081, 0.202]	0.081	[0.018, 0.141]

*Note.* The parameters marked A^W^ refer to the autoregressive effect for women (i.e., the carry-over from one year to the next), whereas the parameters marked A^M^ indicate the autoregressive effect for men (also see Figure 1). All parameters were statistically significantly different from zero at *p* < .001. *r*(^B^W_i_, ^B^M_i_) = correlation between the random intercepts for women and men; CI = credibility interval; ACT = Association of Consumers and Taxpayers; NZ First = New Zealand First; Pol. Ori. = political orientation.

**Figure 2.**
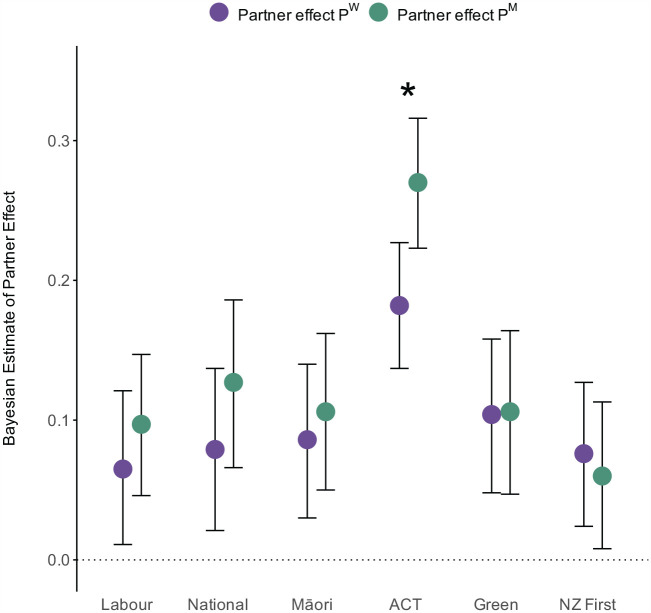
Bayesian estimates and 95% CIs of the unstandardized cross-lagged effects of within-person changes in partners’ political party support on within-person changes in woman and man actors’ political party support (*N*_couples_ = 1,613). All partner effects are significantly different from zero, *p* < .05, except for the partner effects of political orientation. The one significant difference between the partner effects of women and men is marked with an asterisk, indicating that men partners’ effect on women is significantly stronger than vice versa. CI = credibility interval.

Table 4 and Figure 2 present the cross-lagged or partner effects testing whether within-person changes in intimate partners’ political party support predicted subsequent within-person changes in actors’ party support (controlling for the significant similarities in between-person differences in party support across couple members and the autoregressive within-person actor effects). Significant partner (cross-lagged) effects emerged for both women and men across all six political parties: On average, women and men partners who were more supportive of a specific political party at one timepoint significantly predicted actors exhibiting more support for that specific party the subsequent year. The results in Table 4 illustrate the unstandardized effects across all waves. Standardized effects per wave and average standardized effects across waves for each political party are reported in Tables S3 (women) and S4 (men) in the OSM. The average effects for women varied from *β* = .064 to .174, and the average effects for men varied from *β* *=* .068 to .289. According to guidelines for standardized cross-lagged effects, the average effects for women and men were medium to large (Orth et al., 2022).

**Table 4.** Bayesian Estimates of the Unstandardized Cross-Lagged Associations Testing the Effect of Within-Person Changes in Partners’ Political Party Support on Within-Person Changes in Women and Men Actors’ Political Party Support (*N*_couples_ = 1,613).

	P^W^			P^M^				
Outcome	*b*	*p*	95% CI	*b*	*p*	95% CI	Wald (*df*)	*p*
Labour	0.065	.009	[0.011, 0.121]	0.097	<.001	[0.046, 0.147]	0.956 (1)	.328
National	0.079	<.001	[0.021, 0.137]	0.127	<.001	[0.066, 0.186]	2.263 (1)	.133
Māori	0.086	.001	[0.030, 0.140]	0.106	<.001	[0.050, 0.162]	0.398 (1)	.528
ACT	0.182	<.001	[0.137, 0.227]	0.270	<.001	[0.223, 0.316]	10.576 (1)	.001
Green	0.104	<.001	[0.048, 0.158]	0.106	<.001	[0.047, 0.164]	0.005 (1)	.947
NZ First	0.076	.002	[0.024, 0.127]	0.060	.012	[0.008, 0.113]	0.248 (1)	.619
Pol. Ori.	−0.002	.477	[−0.055, 0.051]	0.022	.211	[−0.034, 0.075]	0.496 (1)	.481

*Note.* The parameters marked P^W^ refer to the effect of women partners on men actors, whereas the parameters marked P^M^ indicate the effect of men partners on women actors (also see Figure 1). Wald tests assess gender differences by testing equality constraints on the cross-lagged effects between women and men. The one significant Wald test indicates that P^W^ = P^M^ is statistically significantly different from zero at *p* = .001, meaning that the effect of men partners on women’s ACT party support is larger than the reverse. The intercorrelations of women’s and men’s random intercepts and the autoregressive paths for women (A^W^) and men (A^M^) are available in Table 3. CI = credibility interval; ACT = Association of Consumers and Taxpayers; NZ First = New Zealand First; Pol. Ori. = political orientation.

Finally, we tested whether the partner (cross-lagged) effect (P^W^) of women partners’ political party support on men actors’ political party support was equal to the cross-lagged effect (P^M^) of men partners on women actors’ political party support for each of the six political parties. As shown in the final column of Table 4, Wald tests of the equality across partner (cross-lagged) effects revealed no significant differences between women and men, with one exception: Men’s support of the ACT party had a significantly larger effect on women’s subsequent support for the ACT party than vice versa.

### Intimate Partner Transmission of Political Orientation

In additional analyses, we ran an analogous model to examine whether within-person changes in partners’ political orientation predicted subsequent within-person changes in actors’ political orientation. The significant correlation between the random intercepts for women and men revealed that women and men within intimate relationships had similar political orientations (see Table 3). Although there were significant autoregressive (actor) effects for political orientation (see Table 3), the partner (cross-lagged) effects were not significant (see Table 4). Thus, in contrast to political party support, there was no evidence that intimate partners’ political orientation influenced actors’ political orientation over time.

## Discussion

In an era of heightened political polarization, it is increasingly important to understand how political attitudes change in adulthood (Iyengar et al., 2018). Intimate relationships should be a key context for the transmission of political preferences in which intimate partners may reciprocally influence each other’s support for different political parties over the course of their relationship. However, the degree to which partners’ political preferences predict change in the other’s political preferences across time has not been adequately tested. We overcame limitations in prior couple-based studies by drawing upon large-scale longitudinal panel data to differentiate between-person differences and annual within-person changes in political preferences within 1,613 woman-man couples. Results from APIMs within an RI-CLPM framework provided evidence that within-person increases in intimate partners’ political party support predicted within-person increases in both women and men actors’ party support the following year. These results provide longitudinal evidence that women and men partners mutually influence each other’s support for six political parties across the liberal-conservative ideological spectrum. In the following sections, we describe how these tests provide novel evidence for the transmission of political preferences within intimate relationships, advance the political socialization literature, have important implications, and establish crucial foundations for future research.

### Advances and Implications

Advancing the dominant focus on parent-child relationships in the political socialization literature, the current study emphasizes the importance of adult intimate relationships as a context for the transmission of political preferences. Existing research examining links across intimate partners has primarily involved cross-sectional associations. Although these studies demonstrate similarity in partners’ political preferences, they are unable to distinguish whether similarity emerges because people choose politically aligned partners (assortative mating), they pair with partners from similar backgrounds (social homogamy; Gordon et al., 2024), or couples in established relationships continue to influence each other’s preferences over time. Moreover, the few longitudinal studies attempting to test changes in political preferences within intimate relationships have not used methods that account for stable between-person differences within and between couples. Advancing the methods applied to examine the transmission of political preferences, we conducted APIMs within an RI-CLPM framework to differentiate couple-level associations in trait-like political party support from within-person year-to-year changes in party support. Our results demonstrate that, independent of between-person similarity, within-person changes in intimate partners’ political party support were associated with subsequent within-person changes in actors’ party support. Moreover, assessing these longitudinal partner effects in a multi-party system illustrated that intimate partners’ party support predicted within-person changes in actors’ party support across liberal, moderate, and conservative major and minor political parties. Thus, contrary to past cross-sectional work on major parties, our results provide longitudinal evidence that intimate partners influence each other’s support for various specific parties across the ideological spectrum.

Our results also emphasize the reality of reciprocal transmission of political preferences within adult relationships by revealing symmetrical cross-lagged effects of intimate partners’ support on actors’ political party support across women and men. Despite narratives that men partners likely have a larger impact based on “conventional wisdom” (Stoker & Jennings, 2005) or greater financial power (Kan & Heath, 2006), prior studies have not provided clear evidence of gender differences. The current results provide the strongest evidence to date that gender-based narratives overestimate men’s—and underestimate women’s—roles as agents of political socialization. Indeed, the strength of the partner effect on within-person changes in party support was equal for women and men for five of six tests. One exception indicated that the effect of men partners’ party support on women’s subsequent support for ACT—a libertarian party—was slightly stronger than the effect of women on men’s support. This exception could be due to processes specific to conservative ideologies (e.g., traditional gender roles), but firm conclusions require replication and tests of potential mechanisms. The more consistent message to emerge from our study is that within-person changes in women’s and men’s political party support at one time point equally predicted within-person changes in the other’s party support a year later. Thus, both women and men are likely agents of political socialization within intimate relationships.

Several scholars warn of detrimental societal consequences arising from political homophily within close social relationships (Gordon et al., 2024; Iyengar et al., 2018). For example, individuals from U.S. families who identify with the same political party express more extreme attitudes and more antagonism toward political opponents (Iyengar, 2022). Transmission of political attitudes among intimate partners may also contribute to political polarization. Indeed, research in the United States indicates that intimate partners are more similar to each other than they were 50 years ago (Iyengar et al., 2018). For example, less than 1 in 10 couples now consists of two people who support political parties on opposite sides of the ideological spectrum (Gordon et al., 2024). The transmission of political preferences across intimate partners documented here could result in couples reinforcing their already similar political attitudes, leading to greater political homophily and potentially contributing to polarization. A stronger focus in the literature on adult-to-adult socialization is needed to test this possibility.

### Strengths, Caveats, and Future Research Directions

The current study had several strengths. By leveraging unique dyadic data from a large sample of couples in long-term intimate partnerships, we applied robust statistical methods to isolate within-person changes in political party support over time. Our results reveal that within-person changes in intimate partners’ political party support are longitudinally related to within-person changes in actors’ party support. These longitudinal partner effects emerged across six distinct political parties, indicating that intimate partners may influence each other’s support of various liberal, moderate, and conservative political parties. Although the partner effects may appear small, they align with other longitudinal research that generally observes smaller effect sizes than cross-sectional correlational or experimental studies, especially when using an RI-CLPM to adjust for trait-like differences that persist over time. Taking these considerations into account, the average partner effects observed in the current study qualify as medium to large cross-lagged effects (see Orth et al., 2022). Together, the stringent tests and demonstration of partner effects across six political parties provide strong longitudinal evidence that intimate partners likely influence each other’s political party support.

By revealing partner effects across six political parties in an ideologically diverse multi-party system, the current study also extended previous research on parent-to-child and intimate partner transmission in the U.S. and U.K. political systems (Kan & Heath, 2006; Stoker & Jennings, 2005; Zuckerman et al., 2005). It is especially important to study partner effects in multi-party systems because these are the dominant democratic models worldwide (Green et al., 2002) and because processes that have relatively small effects on political party support, such as intimate partners’ preferences, may lead to little change in overall support or vote choice in two-party systems where political parties are on opposite sides of the ideological spectrum. By contrast, the potential ongoing influence of intimate partners may lead to shifts in voting choice in a system where multiple parties represent similar political agendas. Nonetheless, future research is needed to test the generalizability of our results to other political and national contexts.

The current results also need to be interpreted with the sample and location of the study in mind, which may not generalize to other couples or political contexts. One of the key criteria used to identify couples in the NZAVS was a shared address. Accordingly, we investigated the transmission of political party support in predominantly married cohabiting couples who were already involved in long-term relationships upon entering the study. Transmission across intimate partner preferences may be especially strong in this couple context. For example, compared to non-cohabiting couples, cohabiting couples likely have more opportunities to discuss politics on a regular basis in face-to-face interactions (Coffé & Need, 2010). Potential transmission of political preferences may also be stronger in long-term relationships as intimate partners may know each other more deeply and detect changes in preferences more easily. Moreover, as in most research examining established relationships, these long-term couples reported high levels of relationship satisfaction (see Table S5 in the OSM). Investment in long-term, highly satisfied relationships may amplify the motivation for mutual understanding and shared reality (Jost et al., 2008), resulting in couples being more willing to adapt to each other’s political viewpoints.

Given that our sample consisted of couples within established, long-term relationships, we cannot distinguish between selection and socialization processes at the start of a relationship. For example, more similar partners may be more likely to initiate a relationship but also more likely to establish ongoing relationships that persist across time. Couples who are dissimilar at the outset and/or couples who encounter conflict and dissatisfaction may be less likely to influence each other’s preferences and less likely to maintain their relationship (also see van Scheppingen et al., 2025). Testing these possibilities requires large samples that assess political preferences before (or very soon after) relationships are established (Alford et al., 2011). Despite posing a serious methodological challenge, this design would allow researchers to separate selection from socialization effects and test whether initial selection effects increase the persistence of relationships and/or create stronger socialization effects. Alternatively, large longitudinal samples of couples that include both new and long-term relationships would enable tests of whether socialization effects are stronger in invested, long-term relationships or are equally present due to rapid convergence in early relationships (see Alford et al., 2011). Despite the importance of these future directions and regardless of whether selection processes contributed to the initial maintenance of couples’ relationships, our results suggest that ongoing socialization continues to occur in well-established relationships beyond overall between-person similarity in party support.

More research is also needed to understand whether and why intimate partners are more influential for some types of political preferences than others. Although we found consistent evidence for the influence of partners’ political party support across six different parties, within-person changes in partners’ political orientation (ratings from liberal to conservative) did not predict subsequent changes in actors’ political orientation. Prior studies have shown that parent-child similarity in political preferences is stronger for more concrete, affect-laden (e.g., vote choice, party identification) than abstract, ephemeral (e.g., political orientation) outcomes (Jennings, et al. 2009). Little is currently known about why the transmission of political orientation in parent-child and (in our study) couple relationships may be weaker than political party support. Jennings et al. (2009) suggest that perceptual accuracy and information salience likely play a role. In particular, political discussions likely provide clearer, more concrete information about evaluations and support of political parties than more general political orientations. Indeed, couples’ discussion may focus more on specific political issues and parties, thereby offering greater insight into, and a stronger influence on, changes in each other’s party support compared to political orientation. Explicit discussions or stated preferences for political orientation may also be greater in other countries or political contexts, such as two-party systems that tend to emphasize an identity-based (rather than policy-based) understanding of ideology (e.g., see Kalmoe, 2020). The difference across political party support compared to political orientation may also be because of more variability in, and therefore potential to influence, changes in party support across assessments. Political orientation, by contrast, may be more trait-like and, hence, have less within-person variation across time. In general, more research is needed to understand how people influence each other’s political preferences and how these mechanisms may account for differences in more concrete (e.g., party support) versus general (e.g., political orientation) political preferences within and across different national contexts.

Identifying other conditions that strengthen or weaken intimate partner transmission of party support is also an important future direction. The partner effects observed in the current study show that, on average, women partners’ political party support predicts changes in men actors’ political party support and vice versa. Our analyses use all the data across couples to estimate the average actor and partner effect, but effects will vary according to dynamics within particular couples. For example, educated couples may be more likely to explicitly discuss political party preferences, providing the opportunity to influence each other. Differences within couples could also play an important role. For example, politically engaged partners (Kan & Heath, 2006) or partners who hold more socioeconomic power due to their education, occupation, or financial resources (Mancosu & Vezzoni, 2018) may have a greater influence in shaping actors’ political preferences than partners who are less politically engaged or who have less socioeconomic power. Although people most often discuss politics with their intimate partner compared to friends and family (Carlson et al., 2020), couples will also vary in how much they engage in politically relevant discussions and interactions (which may depend on their similarity). Strong between-person similarity in political preferences within long-term relationships may reveal partner effects that reflect small ongoing changes as couples routinely encounter and discuss politically relevant topics and events. Lower similarity, however, may induce conflict and reactance, leading couples to avoid contentious discussions if they wish to sustain their relationship. Although our stationarity analyses showed that the partner effects did not differ from year to year in this sample, couples’ politically relevant discussions and interactions also may increase in response to salient political events, such as elections, or during particularly tumultuous times, such as the COVID-19 pandemic, potentially amplifying transmission processes at specific time points (e.g., Hammond & Sibley, 2022; Valentino & Sears, 1998).

Finally, our study involved assessing self-reported political party support. Although self-report measures are unavoidable for large-scale panel data like those collected in the NZAVS, such measures may not fully capture behavioral expressions of party support (Sengupta et al., 2023). Nonetheless, studying support rather than behavior can be advantageous in the context of strategic voting, as voting behavior does not necessarily reflect true levels of support. Future investigations could expand on the current study by assessing both political party support and voting behavior to increase understanding of how partners may influence each other’s political preferences, including behaviors. Indeed, although prior research suggests that party identification is the strongest predictor of vote preferences (Smets & Van Ham, 2013), it is important to assess whether the within-person changes in party support we identified translate into political behavior, such as voting, political campaign donations, or party membership. Such evidence will have important implications given that even small annual within-person changes like the ones identified in the current study could accumulate over time to produce meaningful shifts in party support and voting behavior.

## Conclusion

Extending previous research on the parent-child transmission of political attitudes, we provide longitudinal evidence that the reciprocal transmission of political party support occurs across adults in intimate relationships. Assessing within-person changes in political party support in a large sample of cohabiting couples in long-term relationships revealed that intimate partners tend to be quite similar politically on average, and yet appear to continue to influence each other’s political preferences across time. In particular, within-person changes in intimate partners’ political party support were longitudinally associated with within-person changes in actors’ political party support. These partner effects were similar across gender, indicating that women and men are equal agents of political socialization. We encourage future research to consider the transmission of political preferences across different couples and political contexts, including assessing whether partners’ influence builds upon initial selection processes, contributes to relationship persistence, occurs via politically relevant interactions, varies across political contexts, and leads to changes in political behavior. A deeper consideration of the influence intimate partners likely have on each other’s political preferences will advance understanding of the ongoing socialization of political preferences in adulthood.

## Supplemental Material

sj-docx-1-psp-10.1177_01461672251360302 – Supplemental material for Intimate Partners’ Political Influence: Longitudinal Evidence for the Mutual Transmission of Party Support between Women and Men Supplemental material, sj-docx-1-psp-10.1177_01461672251360302 for Intimate Partners’ Political Influence: Longitudinal Evidence for the Mutual Transmission of Party Support between Women and Men by Sam Fluit, Nickola C. Overall, Danny Osborne, Matthew D. Hammond and Chris G. Sibley in Personality and Social Psychology Bulletin
