# Supplementary material for: Intimate Partners’ Political Influence: Longitudinal Evidence for the Mutual Transmission of Party Support between Women and Men
Source: Pers Soc Psychol Bull. 2025 Aug 26;52(9):3080–93. doi: 10.1177/01461672251360302 (PMC13392169; doi:10.1177/01461672251360302)
Supplement: sj-docx-1-psp-10.1177_01461672251360302 – Supplemental material for Intimate Partners’ Political Influence: Longitudinal Evidence for the Mutual Transmission of Party Support between Women and Men [file sj-docx-1-psp-10.1177_01461672251360302.docx]

**Online Supplementary Materials**

These are the online supplementary materials for:

**Intimate Partners’ Political Influence: Longitudinal Evidence for the Mutual Transmission of Party Support between Women and Men**

Sam Fluit, Nickola C. Overall, Danny Osborne, Matthew D. Hammond, & Chris G. Sibley

Contents

[Controlling for relationship length 2](#_Toc196994498)

[Testing for stationarity across time 4](#_Toc196994499)

[Standardized effects at each wave to assess effect size 6](#_Toc196994500)

[Relationship satisfaction across waves 11](#_Toc196994501)

## Controlling for relationship length

Most couples were in long-term married intimate partner relationships. Weighted on the number of couples per wave, the average relationship length was over two and a half decades, *M* = 25.19 years (*SD* = 13.42 years). To account for the possibility of relationship length influencing the partner effects of political party support, we created a new dyad-level variable to assess average relationship length at the mid-point of the annual waves assessed (Time 10; see Table 1 in the main manuscript). To do this, we first calculated relationship length at each annual wave by averaging each partner’s reported relationship length in years (partners reported very similar relationship lengths). Then, to ensure we had a reliable estimate of relationship length at the mid-point of the study for each dyad, we (a) created an estimate of dyad-level relationship length at each wave aligned at T10 (e.g., length_at_T09 + 1; length_at_T11 – 1), and (c) averaged those aligned lengths to give an overall estimate of relationship length at it was or would have been at T10 (in case of non-participation at T10). This estimate was highly reliable in a Confirmatory Factor Analysis of aligned relationship lengths across all timepoints (omega = .999).

To assess the transmission of political preferences controlling for relationship length, we reran the RI-CLPMs regressing relationship length on the observed within-person measures (each individual’s political party support) at each timepoint and constraining those regression parameters to equality separately for women and men. The autoregressive and cross-lagged (partner) paths were estimated as in the primary analyses, with the results estimating the autoregressive and cross-lagged (partner) paths removing any variance related to relationship length. Relationship length had no significant effects, and the results of the associations between partners’ political party support and within-person changes in women and men actors’ political party support remained similar. See Table S1 which presents the Bayesian estimates of the unstandardized cross-lagged effects associations testing the effect of within-person changes in partners’ political party support on within-person changes in women and men actors’ political party support controlling for relationship length.

**Table S1**

*Bayesian estimates of the unstandardized cross-lagged associations testing the effect of within-person changes in partners’ political party support on within-person changes in women and men actors’ political party support controlling for relationship length (N_couples_ = 1,613)*

|  | P^W^ | | | P^M^ | | |  |  |
| --- | --- | --- | --- | --- | --- | --- | --- | --- |
| Outcome | *b* | *p* | 95% CI | *b* | *p* | 95% CI | Wald(df) | *p* |
| Labour | 0.076 | .006 | 0.019 - 0.135 | 0.103 | < .001 | 0.050 - 0.156 | 0.652(1) | .419 |
| National | 0.069 | .008 | 0.013 - 0.127 | 0.124 | < .001 | 0.065 - 0.183 | 2.672(1) | .102 |
| Māori | 0.081 | .002 | 0.024 - 0.134 | 0.104 | < .001 | 0.049 - 0.156 | 0.536(1) | .464 |
| ACT | 0.183 | < .001 | 0.136 - 0.230 | 0.265 | < .001 | 0.216 - 0.314 | 8.809(1) | .003 |
| Green | 0.095 | .001 | 0.037 - 0.152 | 0.101 | < .001 | 0.042 - 0.161 | 0.031(1) | .861 |
| NZ First | 0.073 | .003 | 0.019 - 0.126 | 0.065 | .008 | 0.012 - 0.119 | 0.058(1) | .809 |
| Pol. Ori. | -0.004 | .450 | -0.033 - 0.075 | 0.021 | .222 | -0.033 - 0.075 | 0.539(1) | .463 |

*Note.* NZ First = New Zealand First. Pol. Ori. = Political Orientation. CI = Credibility Interval. The parameters marked P^W^ refer to the effect of women partners on men actors whereas the parameters marked P^M^ indicate the effect of men partners on women actors (also see Figure 1 in the main manuscript). Wald tests assess gender differences by testing equality constraints on the cross-lagged effects between women and men. The one significant Wald test indicates that P^W^ = P^M^ is statistically significantly different from zero at *p* = .001 meaning that the effect of men partners on women’s ACT party support is larger than the reverse.

## Testing for stationarity across time

Although there were no reasons to expect the autoregressive (actor) and cross-lagged (partner) paths to vary across assessments (see Osborne & Little, 2024), we tested our assumption of stationarity across waves with a series of nested models (see Table S2). First, we estimated fully unconstrained nonstationary models that allowed both autoregressive and cross-lagged (partner) paths to vary across waves (full nonstationary model, top row Table S2). Second, we fitted partially stationary models in which we constrained autoregressive (actor) paths to be equal across waves but allowed the cross-lagged (partner) paths to vary (partially stationary model, middle row Table S2). Third, we estimated fully stationary models that constrained both autoregressive (actor) and cross-lagged (partner) paths to be equal across waves (stationary model, bottom row Table S2). As can be seen in Table S2, the stationary models provided the best model fit and represent the most parsimonious solution for all outcomes tested. Thus, in the primary analyses, we constrained the autoregressive (actor) and cross-lagged (partner) paths to be equal across waves. The Bayesian estimates of autoregressive and cross-lagged (partner) paths from the stationary model are reported in the main manuscript.

**Table S2**

*Model fit for full nonstationary, partially stationary, and stationary Bayesian estimation of the effect of within-person changes in partners’ political party support on within-person changes in women and men actors’ political party support (N_couples_ = 1,613)*

|  | Full nonstationary model | | | | | |
| --- | --- | --- | --- | --- | --- | --- |
| Outcome | 95% CI(Δχ²) | BIC | DIC | RMSEA | CFI | TLI |
| Labour | [-4.690-120.257] | 29,951.395 | 29,470.341 | 0.000 | 1.000 | 1.000 |
|  |  |  |  |  |  |  |
| National | [13.248-139.147] | 29,757.841 | 29,276.775 | 0.000 | 1.000 | 1.000 |
|  |  |  |  |  |  |  |
| Māori | [7.931-130.921] | 30,567.734 | 30,086.909 | 0.000 | 1.000 | 1.000 |
|  |  |  |  |  |  |  |
| ACT | [19.597-144.392] | 30,990.658 | 30,509.010 | 0.000 | 1.000 | 1.000 |
|  |  |  |  |  |  |  |
| Green | [12.515-136.825] | 30,333.547 | 29,852.303 | 0.000 | 1.000 | 1.000 |
|  |  |  |  |  |  |  |
| NZ First | [-22.890-99.918] | 30,108.390 | 29,627.097 | 0.000 | 1.000 | 1.000 |
|  |  |  |  |  |  |  |
| Pol. Ori. | [-36.170-86.176] | 26,438.730 | 25,957.179 | 0.000 | 1.000 | 1.000 |
|  | Partially stationary model | | | | | |
| Outcome | 95% CI(Δχ²) | BIC | DIC | RMSEA | CFI | TLI |
| Labour | [41.932-163.138] | 29,902.053 | 29,506.831 | 0.000 | 1.000 | 1.000 |
|  |  |  |  |  |  |  |
| National | [61.099-187.777] | 29,719.891 | 29,324.808 | 0.000 | 1.000 | 1.000 |
|  |  |  |  |  |  |  |
| Māori | [63.432-185.611] | 30,526.820 | 30,130.727 | 0.000 | 1.000 | 1.000 |
|  |  |  |  |  |  |  |
| ACT | [61.639-186.644] | 30,946.557 | 30,551.471 | 0.000 | 1.000 | 1.000 |
|  |  |  |  |  |  |  |
| Green | [23.757-149.729] | 30,259.367 | 29,864.705 | 0.000 | 1.000 | 1.000 |
|  |  |  |  |  |  |  |
| NZ First | [18.052-141.007] | 30,054.329 | 29,659.126 | 0.000 | 1.000 | 1.000 |
|  |  |  |  |  |  |  |
| Pol. Ori. | [-33.277-88.175] | 26,337.290 | 25,942.221 | 0.000 | 1.000 | 1.000 |
|  | Stationary model | | | | | |
| Outcome | 95% CI(Δχ²) | BIC | DIC | RMSEA | CFI | TLI |
| Labour | [55.372-178.023] | 29,810.559 | 29,501.813 | 0.000 | 1.000 | 1.000 |
|  |  |  |  |  |  |  |
| National | [63.458-190.522] | 29,621.644 | 29,312.446 | 0.000 | 1.000 | 1.000 |
|  |  |  |  |  |  |  |
| Māori | [100.990-225.237] | 30,470.944 | 30,162.228 | 0.000 | 1.000 | 1.000 |
|  |  |  |  |  |  |  |
| ACT | [140.486-264.794] | 30,918.872 | 30,611.486 | 0.000 | 1.000 | 1.000 |
|  |  |  |  |  |  |  |
| Green | [26.720-150.499] | 30,159.856 | 29,851.551 | 0.000 | 1.000 | 1.000 |
|  |  |  |  |  |  |  |
| NZ First | [25.162-145.540] | 29,957.883 | 29,649.475 | 0.000 | 1.000 | 1.000 |
|  |  |  |  |  |  |  |
| Pol. Ori. | [-33.942-88.469] | 26,236.527 | 25,928.289 | 0.000 | 1.000 | 1.000 |

*Note*. 95% CI(Δχ²) = 95% Confidence Interval for the Difference Between Observed and Replicated Chi-Square Values. BIC = Bayesian Information Criterion. DIC = Deviance Information Criterion. RMSEA = Root Mean Square Error of Approximation. CFI = Comparative Fit Index. TLI = Tucker-Lewis Index. NZ First = New Zealand First. Pol. Ori. = Political Orientation.

## Standardized effects at each wave to assess effect size

To evaluate the strength of the partner effect, we calculated standardized effects per wave per political party for women (see Table S3) and men (see Table S4). The model constrained both autoregressive (actor) and cross-lagged (partner) paths to be equal across waves but did not constrain the variances to be equal at each wave. This means that, although the unstandardized effect reported in the paper is the same across waves, the standardized effects that express the effect size in terms of standard deviations will vary according to any differences in the variances at each wave. We compared the mean standardized effects across waves per political party to the guidelines for standardized effects in RI-CLPMs by Orth et al. (2022). The average effects across political parties represented medium to large effects for both women (.064 ≤ *β* ≤ .174; see column *Mean* in Table S3) and men (.068 ≤ *β* ≤. 289; see column *Mean* in Table S4).

**Table S3**

*Standardized autoregressive and cross-lagged effects for women*

|  |  | Time 6 | | |  | Time 7 | | |  | Time 8 | | |  | Time 9 | | |  | Time 10 | | |
| --- | --- | --- | --- | --- | --- | --- | --- | --- | --- | --- | --- | --- | --- | --- | --- | --- | --- | --- | --- | --- |
|  |  |  | 95% CI | |  |  | 95% CI | |  |  | 95% CI | |  |  | 95% CI | |  |  | 95% CI | |
| Outcome | Predictor_T-1_ | β | Lower | Upper |  | β | Lower | Upper |  | β | Lower | Upper |  | β | Lower | Upper |  | β | Lower | Upper |
| Labour | A^W^ | .320^***^ | [.237, | .414] |  | .415^***^ | [.312, | .528] |  | .325^***^ | [.241, | .424] |  | .308^***^ | [.240, | .386] |  | .402^***^ | [.311, | .498] |
|  | P^W^ | .067^**^ | [.012, | .130] |  | .068^**^ | [.011, | .133] |  | .064^**^ | [.011, | .123] |  | .056^**^ | [.010, | .107] |  | .073^**^ | [.012, | .138] |
|  |  |  |  |  |  |  |  |  |  |  |  |  |  |  |  |  |  |  |  |  |
| National | A^W^ | .307^***^ | [.220, | .413] |  | .290^***^ | [.213, | .382] |  | .343^***^ | [.247, | .461] |  | .284^***^ | [.213, | .366] |  | .320^***^ | [.234, | .425] |
|  | P^W^ | .098^**^ | [.027, | .172] |  | .094^**^ | [.025, | .174] |  | .088^**^ | [.023, | .161] |  | .076^**^ | [.020, | .137] |  | .082^**^ | [.021, | .153] |
|  |  |  |  |  |  |  |  |  |  |  |  |  |  |  |  |  |  |  |  |  |
| Māori | A^W^ | .209^***^ | [.134, | .303] |  | .213^***^ | [.134, | .301] |  | .201^***^ | [.126, | .294] |  | .179^***^ | [.117, | .247] |  | .252^***^ | [.161, | .355] |
|  | P^W^ | .094^**^ | [.032, | .161] |  | .085^**^ | [.030, | .144] |  | .088^**^ | [.030, | .150] |  | .076^**^ | [.026, | .127] |  | .096^**^ | [.033, | .161] |
|  |  |  |  |  |  |  |  |  |  |  |  |  |  |  |  |  |  |  |  |  |
| ACT | A^W^ | .380^***^ | [.287, | .482] |  | .404^***^ | [.322, | .487] |  | .430^***^ | [.357, | .509] |  | .407^***^ | [.338, | .476] |  | .485^***^ | [.411, | .562] |
|  | P^W^ | .168^***^ | [.114, | .233] |  | .170^***^ | [.123, | .222] |  | .194^***^ | [.142, | .249] |  | .173^***^ | [.126, | .221] |  | .194^***^ | [.143, | .245] |
|  |  |  |  |  |  |  |  |  |  |  |  |  |  |  |  |  |  |  |  |  |
| Green | A^W^ | .158^***^ | [.095, | .241] |  | .188^***^ | [.111, | .283] |  | .150^***^ | [.089, | .224] |  | .187^***^ | [.116, | .269] |  | .169^***^ | [.102, | .245] |
|  | P^W^ | .134^***^ | [.060, | .219] |  | .103^***^ | [.046, | .164] |  | .095^***^ | [.043, | .152] |  | .102^***^ | [.046, | .162] |  | .106^***^ | [.048, | .170] |
|  |  |  |  |  |  |  |  |  |  |  |  |  |  |  |  |  |  |  |  |  |
| NZ First | A^W^ | .146^***^ | [.088, | .216] |  | .147^***^ | [.088, | .217] |  | .190^***^ | [.115, | .283] |  | .138^***^ | [.083, | .203] |  | .188^***^ | [.115, | .268] |
|  | P^W^ | .063^**^ | [.019, | .111] |  | .067^**^ | [.021, | .119] |  | .073^**^ | [.023, | .128] |  | .064^**^ | [.020, | .112] |  | .084^**^ | [.026, | .145] |
|  |  |  |  |  |  |  |  |  |  |  |  |  |  |  |  |  |  |  |  |  |
| Pol. Ori. | A^W^ | .160^***^ | [.091, | .247] |  | .113^***^ | [.062, | .178] |  | .170^***^ | [.095, | .266] |  | .130^***^ | [.074, | .197] |  | .145^***^ | [.082, | .225] |
|  | P^W^ | -.001 | [-.053, | .048] |  | -.001 | [-.051, | .049] |  | -.002 | [-.067, | .062] |  | -.002 | [-.057, | .053] |  | -.002 | [-.062, | .058] |

*Note*. CI = Credibility Interval. A^W^ = Autoregressive paths for women. P^W^ = Women-on-men partner paths. NZ First = New Zealand First. Pol. Ori. = Political Orientation.

^*^*p <* .05, ^**^*p <* .01, ^***^*p <* .001.

**Table S3** (continued)

*Standardized autoregressive and cross-lagged effects for women*

|  |  | Time 11 | | |  | Time 12 | | |  | Time 13 | | |  | Time 14 | | | Mean |
| --- | --- | --- | --- | --- | --- | --- | --- | --- | --- | --- | --- | --- | --- | --- | --- | --- | --- |
|  |  |  | 95% CI | |  |  | 95% CI | |  |  | 95% CI | |  |  | 95% CI | |  |
| Outcome | Predictor_T-1_ | β | Lower | Upper |  | β | Lower | Upper |  | β | Lower | Upper |  | β | Lower | Upper | β |
| Labour | A^W^ | .364^***^ | [.300, | .429] |  | .305^***^ | [.246, | .367] |  | .359^***^ | [.291, | .425] |  | .408^***^ | [.329, | .487] | .356 |
|  | P^W^ | .062^**^ | [.011, | .116] |  | .059^**^ | [.010, | .110] |  | .066^**^ | [.011, | .123] |  | .065^**^ | [.011, | .122] | .064 |
|  |  |  |  |  |  |  |  |  |  |  |  |  |  |  |  |  |  |
| National | A^W^ | .326^***^ | [.260, | .393] |  | .293^***^ | [.226, | .361] |  | .333^***^ | [.258, | .406] |  | .315^***^ | [.241, | .393] | .312 |
|  | P^W^ | .083^**^ | [.023, | .144] |  | .081^**^ | [.022, | .143] |  | .080^**^ | [.021, | .141] |  | .088^**^ | [.023, | .154] | .086 |
|  |  |  |  |  |  |  |  |  |  |  |  |  |  |  |  |  |  |
| Māori | A^W^ | .199^***^ | [.133, | .266] |  | .207^***^ | [.140, | .275] |  | .215^***^ | [.141, | .291] |  | .203^***^ | [.131, | .279] | .210 |
|  | P^W^ | .080^**^ | [.028, | .131] |  | .079^**^ | [.028, | .128] |  | .082^**^ | [.028, | .135] |  | .084^**^ | [.029, | .138] | .085 |
|  |  |  |  |  |  |  |  |  |  |  |  |  |  |  |  |  |  |
| ACT | A^W^ | .371^***^ | [.316, | .427] |  | .386^***^ | [.333, | .439] |  | .430^***^ | [.371, | .487] |  | .448^***^ | [.384, | .508] | .419 |
|  | P^W^ | .146^***^ | [.107, | .187] |  | .164^***^ | [.121, | .208] |  | .177^***^ | [.131, | .222] |  | .178^***^ | [.132, | .224] | .174 |
|  |  |  |  |  |  |  |  |  |  |  |  |  |  |  |  |  |  |
| Green | A^W^ | .182^***^ | [.117, | .249] |  | .172^***^ | [.107, | .239] |  | .173^***^ | [.108, | .241] |  | .157^***^ | [.095, | .225] | .198 |
|  | P^W^ | .105^***^ | [.048, | .161] |  | .098^***^ | [.045, | .152] |  | .103^***^ | [.047, | .160] |  | .100^***^ | [.046, | .155] | .105 |
|  |  |  |  |  |  |  |  |  |  |  |  |  |  |  |  |  |  |
| NZ First | A^W^ | .164^***^ | [.103, | .228] |  | .180^***^ | [.115, | .245] |  | .182^***^ | [.112, | .255] |  | .156^***^ | [.096, | .222] | .179 |
|  | P^W^ | .073^**^ | [.023, | .125] |  | .084^**^ | [.027, | .143] |  | .076^**^ | [.024, | .130] |  | .069^**^ | [.022, | .118] | .073 |
|  |  |  |  |  |  |  |  |  |  |  |  |  |  |  |  |  |  |
| Pol. Ori. | A^W^ | .155^***^ | [.091, | .224] |  | .133^***^ | [.077, | .196] |  | .143^***^ | [.082, | .211] |  | .139^***^ | [.079, | .204] | .159 |
|  | P^W^ | -.002 | [-.059, | .055] |  | -.002 | [-.053, | .050] |  | -.002 | [-.053, | .050] |  | -.001 | [-.051, | .047] | -.002 |

*Note*. CI = Credibility Interval. A^W^ = Autoregressive paths for women. P^W^ = Women-on-men partner paths. NZ First = New Zealand First. Pol. Ori. = Political Orientation.

Mean refers to the average standardized effect across waves.

^*^*p <* .05, ^**^*p <* .01, ^***^*p <* .001.

**Table S4**

*Standardized autoregressive and cross-lagged effects for men*

|  |  | Time 6 | | |  | Time 7 | | |  | Time 8 | | |  | Time 9 | | |  | Time 10 | | |
| --- | --- | --- | --- | --- | --- | --- | --- | --- | --- | --- | --- | --- | --- | --- | --- | --- | --- | --- | --- | --- |
|  |  |  | 95% CI | |  |  | 95% CI | |  |  | 95% CI | |  |  | 95% CI | |  |  | 95% CI | |
| Outcome | Predictor_T-1_ | β | Lower | Upper |  | β | Lower | Upper |  | β | Lower | Upper |  | β | Lower | Upper |  | β | Lower | Upper |
| Labour | A^M^ | .402^***^ | [.300, | .515] |  | .297^***^ | [.214, | .399] |  | .355^***^ | [.269, | .453] |  | .259^***^ | [.193, | .336] |  | .367^***^ | [.280, | .462] |
|  | P^M^ | .108^***^ | [.049, | .174] |  | .102^***^ | [.047, | .165] |  | .102^***^ | [.047, | .162] |  | .081^***^ | [.037, | .129] |  | .115^***^ | [.054, | .179] |
|  |  |  |  |  |  |  |  |  |  |  |  |  |  |  |  |  |  |  |  |  |
| National | A^M^ | .259^***^ | [.178, | .358] |  | .214^***^ | [.138, | .315] |  | .193^***^ | [.128, | .278] |  | .215^***^ | [.151, | .293] |  | .218^***^ | [.144, | .315] |
|  | P^M^ | .117^***^ | [.059, | .183] |  | .094^***^ | [.047, | .151] |  | .108^***^ | [.053, | .170] |  | .114^***^ | [.059, | .173] |  | .122^***^ | [.061, | .196] |
|  |  |  |  |  |  |  |  |  |  |  |  |  |  |  |  |  |  |  |  |  |
| Māori | A^M^ | .393^***^ | [.303, | .499] |  | .328^***^ | [.252, | .412] |  | .374^***^ | [.286, | .468] |  | .299^***^ | [.234, | .376] |  | .388^***^ | [.306, | .480] |
|  | P^M^ | .107^***^ | [.050, | .175] |  | .099^***^ | [.046, | .161] |  | .104^***^ | [.048, | .170] |  | .086^***^ | [.040, | .136] |  | .124^***^ | [.058, | .194] |
|  |  |  |  |  |  |  |  |  |  |  |  |  |  |  |  |  |  |  |  |  |
| ACT | A^M^ | .499^***^ | [.388, | .612] |  | .506^***^ | [.424, | .592] |  | .608^***^ | [.532, | .680] |  | .506^***^ | [.428, | .583] |  | .605^***^ | [.534, | .675] |
|  | P^M^ | .230^***^ | [.168, | .310] |  | .247^***^ | [.194, | .305] |  | .277^***^ | [.224, | .336] |  | .246^***^ | [.195, | .301] |  | .312^***^ | [.255, | .370] |
|  |  |  |  |  |  |  |  |  |  |  |  |  |  |  |  |  |  |  |  |  |
| Green | A^M^ | .355^***^ | [.252, | .474] |  | .208^***^ | [.147, | .285] |  | .299^***^ | [.213, | .396] |  | .278^***^ | [.207, | .355] |  | .338^***^ | [.238, | .450] |
|  | P^M^ | .092^***^ | [.041, | .154] |  | .084^***^ | [.037, | .141] |  | .104^***^ | [.046, | .171] |  | .113^***^ | [.050, | .182] |  | .118^***^ | [.052, | .189] |
|  |  |  |  |  |  |  |  |  |  |  |  |  |  |  |  |  |  |  |  |  |
| NZ First | A^M^ | .251^***^ | [.180, | .338] |  | .240^***^ | [.167, | .323] |  | .252^***^ | [.180, | .339] |  | .258^***^ | [.189, | .336] |  | .281^***^ | [.206, | .363] |
|  | P^M^ | .063^*^ | [.008, | .124] |  | .057^*^ | [.007, | .115] |  | .071^*^ | [.009, | .137] |  | .060^*^ | [.008, | .117] |  | .068^*^ | [.009, | .132] |
|  |  |  |  |  |  |  |  |  |  |  |  |  |  |  |  |  |  |  |  |  |
| Pol. Ori. | A^M^ | .072^*^ | [.016, | .134] |  | .092^*^ | [.021, | .169] |  | .084^*^ | [.019, | .157] |  | .084^*^ | [.019, | .154] |  | .083^*^ | [.019, | .151] |
|  | P^M^ | .024 | [.008, | .086] |  | .022 | [.007, | .078] |  | .023 | [.009, | .084] |  | .021 | [.008, | .074] |  | .021 | [.009, | .074] |

*Note*. CI = Credibility Interval. A^W^ = Autoregressive paths for men. P^W^ = Men-on-women partner paths. NZ First = New Zealand First. Pol. Ori. = Political Orientation.

^*^*p <* .05, ^**^*p <* .01, ^***^*p <* .001.

**Table S4** (continued)

*Standardized autoregressive and cross-lagged effects for men*

|  |  | Time 11 | | |  | Time 12 | | |  | Time 13 | | |  | Time 14 | | | Mean |
| --- | --- | --- | --- | --- | --- | --- | --- | --- | --- | --- | --- | --- | --- | --- | --- | --- | --- |
|  |  |  | 95% CI | |  |  | 95% CI | |  |  | 95% CI | |  |  | 95% CI | |  |
| Outcome | Predictor_T-1_ | β | Lower | Upper |  | β | Lower | Upper |  | β | Lower | Upper |  | β | Lower | Upper | β |
| Labour | A^M^ | .313^***^ | [.252, | .376] |  | .316^***^ | [.249, | .383] |  | .311^***^ | [.244, | .380] |  | .324^***^ | [.251, | .398] | .327 |
|  | P^M^ | .104^***^ | [.049, | .159] |  | .093^***^ | [.044, | .142] |  | .096^***^ | [.046, | .147] |  | .116^***^ | [.054, | .178] | .102 |
|  |  |  |  |  |  |  |  |  |  |  |  |  |  |  |  |  |  |
| National | A^M^ | .235^***^ | [.171, | .302] |  | .229^***^ | [.160, | .306] |  | .206^***^ | [.145, | .278] |  | .262^***^ | [.184, | .347] | .226 |
|  | P^M^ | .132^***^ | [.069, | .193] |  | .118^***^ | [.060, | .176] |  | .123^***^ | [.063, | .183] |  | .135^***^ | [.069, | .200] | .118 |
|  |  |  |  |  |  |  |  |  |  |  |  |  |  |  |  |  |  |
| Māori | A^M^ | .356^***^ | [.295, | .420] |  | .336^***^ | [.278, | .396] |  | .367^***^ | [.302, | .434] |  | .379^***^ | [.306, | .453] | .358 |
|  | P^M^ | .109^***^ | [.052, | .168] |  | .108^***^ | [.052, | .166] |  | .118^***^ | [.055, | .183] |  | .112^***^ | [.052, | .175] | .119 |
|  |  |  |  |  |  |  |  |  |  |  |  |  |  |  |  |  |  |
| ACT | A^M^ | .483^***^ | [.425, | .541] |  | .550^***^ | [.487, | .609] |  | .551^***^ | [.489, | .611] |  | .573^***^ | [.511, | .631] | .542 |
|  | P^M^ | .253^***^ | [.205, | .303] |  | .267^***^ | [.218, | .319] |  | .276^***^ | [.226, | .329] |  | .297^***^ | [.244, | .352] | .289 |
|  |  |  |  |  |  |  |  |  |  |  |  |  |  |  |  |  |  |
| Green | A^M^ | .285^***^ | [.221, | .350] |  | .292^***^ | [.220, | .363] |  | .311^***^ | [.233, | .390] |  | .287^***^ | [.212, | .366] | .295 |
|  | P^M^ | .109^***^ | [.049, | .170] |  | .113^***^ | [.050, | .178] |  | .115^***^ | [.051, | .181] |  | .100^***^ | [.044, | .159] | .116 |
|  |  |  |  |  |  |  |  |  |  |  |  |  |  |  |  |  |  |
| NZ First | A^M^ | .255^***^ | [.191, | .320] |  | .295^***^ | [.226, | .361] |  | .251^***^ | [.185, | .319] |  | .256^***^ | [.190, | .326] | .260 |
|  | P^M^ | .062^*^ | [.008, | .117] |  | .068^*^ | [.009, | .128] |  | .065^*^ | [.008, | .125] |  | .062^*^ | [.008, | .120] | .068 |
|  |  |  |  |  |  |  |  |  |  |  |  |  |  |  |  |  |  |
| Pol. Ori. | A^M^ | .080^*^ | [.019, | .139] |  | .082^*^ | [.019, | .145] |  | .076^*^ | [.017, | .135] |  | .079^*^ | [.018, | .140] | .081 |
|  | P^M^ | .022 | [.008, | .078] |  | .022 | [.009, | .075] |  | .022 | [.008, | .076] |  | .023 | [.008, | .080] | .022 |

*Note*. CI = Credibility Interval. A^W^ = Autoregressive paths for men. P^W^ = Men-on-women partner paths. NZ First = New Zealand First. Pol. Ori. = Political Orientation.

Mean refers to the average standardized effect across waves.

^*^*p <* .05, ^**^*p <* .01, ^***^*p <* .001.

## Relationship satisfaction across waves

Table S5 provides the reported relationship satisfaction for women and men at each wave. Relationship satisfaction was measured with the following question: “How satisfied are you with your relationship?” with answer options ranging from 1 (*Not satisfied*) to 7 (*Very satisfied*). As typical for couples sampled from the community, average relationship satisfaction was high across waves. Weighted on the number of couples in each wave, the averages were: *M*_women_ = 6.16 (*SD*_women_ = 1.19) and *M*_men_ = 6.19 (*SD*_men_ = 1.07). In sum, the sample comprised of cohabitating, mostly married, couples involved in long-term highly satisfied relationships.

**Table S5**

*Relationship satisfaction for women and men across ten consecutive years of data collection*

|  |  | Mean(*SD*) | |
| --- | --- | --- | --- |
| Time | *N* | Women | Men |
| Time 5 | 136 | 6.18(1.11) | 6.20(1.10) |
| Time 6 | 112 | 6.24(1.13) | 6.21(1.02) |
| Time 7 | 93 | 6.25(1.14) | 6.23(1.01) |
| Time 8 | 199 | 6.18(1.07) | 6.19(0.98) |
| Time 9 | 136 | 6.13(1.10) | 6.16(0.86) |
| Time 10 | 874 | 6.14(1.11) | 6.22(1.03) |
| Time 11 | 813 | 6.15(1.14) | 6.19(1.03) |
| Time 12 | 950 | 6.16(1.08) | 6.18(1.06) |
| Time 13 | 721 | 6.13(1.07) | 6.15(1.08) |
| Time 14 | 677 | 6.20(1.01) | 6.19(1.01) |

# References

Orth, U., Meier, L. L., Bühler, J. L., Dapp, L. C., Krauss, S., Messerli, D., & Robins, R. W. (2022). Effect size guidelines for cross-lagged effects. *Psychological Methods*. https://doi.org/10.1037/met0000499

Osborne, D., & Little, T. D. (2024). The random intercepts cross-lagged panel model. In *Longitudinal structural equation modeling* (2nd Edition). The Guilford Press.
